# Complete Genome Sequence of Porcine Epidemic Diarrhea Virus Strain USA/Colorado/2013 from the United States

**DOI:** 10.1128/genomeA.00555-13

**Published:** 2013-08-08

**Authors:** Douglas Marthaler, Yin Jiang, Tracy Otterson, Sagar Goyal, Kurt Rossow, James Collins

**Affiliations:** University of Minnesota Veterinary Diagnostic Laboratory, Saint Paul, Minnesota, USA

## Abstract

Porcine epidemic diarrhea virus (PEDV) is newly emerging in the United States. PEDV strain USA/Colorado/2013 (CO/13) was obtained from a 7-day-old piglet with severe diarrhea, and the complete genome was sequenced to further study the PEDV outbreak in the United States.

## GENOME ANNOUNCEMENT

Porcine epidemic diarrhea virus (PEDV), a member of the *Coronaviridae* family, is an enveloped, positive-sense, single-strand RNA virus that causes severe diarrhea and dehydration in pigs. First identified in Europe in 1971, PEDV was characterized in feeder and fattening pigs and subsequently has been identified in Belgium, Hungary, Korea, Italy, Thailand, Japan, and China (1–10). More recently, PEDV outbreaks in China have been described as more clinically severe than historic strains (1, 11, 12). In the United States, PEDV was identified on 29 April 2013 by the Iowa State University Veterinary Diagnostic Laboratory and was corroborated by the National Veterinary Services Laboratories (NVSL) on 16 May 2013 (http://www.aasv.org/news/story.php?id=6444). To determine the phylogenetic relationship of the new U.S. strain and global PEDV strains, the complete genome of PEDV CO/13 was sequenced and analyzed.

At the University of Minnesota Veterinary Diagnostic Laboratory, a fecal sample from a 7-day-old pig was processed, RNA was extracted, and the sample was found to be positive for PEDV by reverse transcription-PCR (RT-PCR) using previously published methods (2, 13, 14). The RNA was submitted to the University of Minnesota Genomics Center for Illumina TruSeq (Illumina, San Diego, CA) library preparation and next-generation sequencing (NGS) on Illumina MiSeq (Illumina, San Diego, CA). Using a template assembly (SeqMan NGen, DNAStar version 11; Madison, WI), the CO/13 NGS reads were assembled using complete PEDV reference genomes from GenBank (9, 11, 12, 15–23). The GenBank reference strains were removed to create a single sequence of CO/13. Finally, the CO/13 NGS reads were remapped to the initial sequence of CO/13 to verify the accuracy of the initial assembly. The single genomic gap of CO/13 was closed using standard Sanger sequencing technology. The complete genomic sequence of CO/13 is 28,038 nucleotides (nt), excluding the 3′ poly(A) tail. The genome arrangement and corresponding nucleotide positions are as follows: 5′ untranslated region (UTR), nt 1 to 292; replicase, nt 293 to 12646 for 1a, and nt 12601 to 20637 for 1b; spike (S), nt 20634 to 24794; open reading frame 3 (ORF3), nt 24794 to 25468; envelope (E), nt 25449 to 25679; membrane (M), nt 25687 to 26367; nucleocapsid (N), nt 26379 to 27704; and 3′ untranslated region, nt 27706 to 28038.

The complete PEDV genome of CO/13 has a nucleotide identity of 96.5 to 99.5% with other complete PEDV genomes available in GenBank, with the highest nucleotide identity (99.5%) with Chinese strain AH2012 (GenBank accession no. KC210145) (9, 11, 12, 15–23). Comparing the complete genome of CO/13 to that of PEDV reference strain CV777, CO/13 contains a 1-nt insertion (at position 48) and deletions of 5 nt in the 5′ UTR (at positions 73 and 83 to 86), while the spike gene contains insertions of 16 nt (positions 20804, 20810 to 20820, 20843, and 21053 to 21055) and deletions of 7 nt (positions 20853 and 21118 to 21124) (18).

To our knowledge, this is the first complete U.S. PEDV sequence to be released. The PEDV USA/Colorado/2013 sequence data will further our understanding of the genetic diversity of PEDVs and facilitate future research on the epidemiology and evolution of PEDVs in the United States.

### Nucleotide sequence accession number.

The USA/Colorado/2013 PEDV sequence data have been deposited in GenBank under the accession no. KF272920.

## References

[1] Jinghui F Yijing L 2005 Cloning and sequence analysis of the M gene of porcine epidemic diarrhea virus LJB/03. Virus Genes 30:69–73. 10.1007/s11262-004-4583-z 15744564 PMC7089338

[2] Li Z Chen F Yuan Y Zeng X Wei Z Zhu L Sun B Xie Q Cao Y Xue C Ma J Bee Y 2013 Sequence and phylogenetic analysis of nucleocapsid genes of porcine epidemic diarrhea virus (PEDV) strains in China. Arch. Virol. 158:1267–1273. 10.1007/s00705-012-1592-4 23389550 PMC3668129

[3] Wood EN 1977 An apparently new syndrome of porcine epidemic diarrhoea. Vet. Rec. 100:243–244. 10.1136/vr.100.12.243 888300

[4] Song D Park B 2012 Porcine epidemic diarrhoea virus: a comprehensive review of molecular epidemiology, diagnosis, and vaccines. Virus Genes 44:167–175. 10.1007/s11262-012-0713-1 22270324 PMC7089188

[5] Chae C Kim O Choi C Min K Cho WS Kim J Tai JH 2000 Prevalence of porcine epidemic diarrhoea virus and transmissible gastroenteritis virus infection in Korean pigs. Vet. Rec. 147:606–608. 10.1136/vr.147.21.606 11110482

[6] Martelli P Lavazza A Nigrelli AD Merialdi G Alborali LG Pensaert MB 2008 Epidemic of diarrhoea caused by porcine epidemic diarrhoea virus in Italy. Vet. Rec. 162:307–310. 10.1136/vr.162.10.307 18326842

[7] Nagy B Nagy G Meder M Mocsári E 1996 Enterotoxigenic *Escherichia coli*, rotavirus, porcine epidemic diarrhoea virus, adenovirus and calici-like virus in porcine postweaning diarrhoea in Hungary. Acta Vet. Hung. 44:9–19 8826697

[8] Pensaert MB de Bouck P 1978 A new coronavirus-like particle associated with diarrhea in swine. Arch. Virol. 58:243–247. 10.1007/BF01317606 83132 PMC7086830

[9] Park S-J Kim H-K Song D-S An D-J Park B-K 2012 Complete genome sequences of a Korean virulent porcine epidemic diarrhea virus and its attenuated counterpart. J. Virol. 86:5964 5964. 10.1128/JVI.00557-12 22532530 PMC3347302

[10] Puranaveja S Poolperm P Lertwatcharasarakul P Kesdaengsakonwut S Boonsoongnern A Urairong K Kitikoon P Choojai P Kedkovid R Teankum K Thanawongnuwech R 2009 Chinese-like strain of porcine epidemic diarrhea virus, Thailand. Emerg. Infect. Dis. 15:1112–1115. 10.3201/eid1507.081256 19624933 PMC2744260

[11] Chen J Liu X Shi D Shi H Zhang X Feng L 2012 Complete genome sequence of a porcine epidemic diarrhea virus variant. J. Virol. 86:3408 3408. 10.1128/JVI.07150-11 22354946 PMC3302349

[12] Gao Y Kou Q Ge X Zhou L Guo X Yang H 2013 Phylogenetic analysis of porcine epidemic diarrhea virus field strains prevailing recently in China. Arch. Virol. 158:711–715. 10.1007/s00705-012-1541-2 23151819

[13] Marthaler D Rossow K Gramer M Collins J Goyal S Tsunemitsu H Kuga K Suzuki T Ciarlet M Matthijnssens J 2012 Detection of substantial porcine group B rotavirus genetic diversity in the United States, resulting in a modified classification proposal for G genotypes. Virology 433:85–96. 10.1016/j.virol.2012.07.006 22877843 PMC7111968

[14] Li ZL Zhu L Ma JY Zhou QF Song YH Sun BL Chen RA Xie QM Bee YZ 2012 Molecular characterization and phylogenetic analysis of porcine epidemic diarrhea virus (PEDV) field strains in south China. Virus Genes 45:181–185. 10.1007/s11262-012-0735-8 22528639

[15] Bi J Zeng S Xiao S Chen H Fang L 2012 Complete genome sequence of porcine epidemic diarrhea virus strain AJ1102 isolated from a suckling piglet with acute diarrhea in China. J. Virol. 86:10910–10911. 10.1128/JVI.01919-12 22966198 PMC3457323

[16] Chen J Wang C Shi H Qiu H-J Liu S Shi D Zhang X Feng L 2011 Complete genome sequence of a Chinese virulent porcine epidemic diarrhea virus strain. J. Virol. 85:11538–11539. 10.1128/JVI.06024-11 21980030 PMC3194942

[17] Fan H Zhang J Ye Y Tong T Xie K Liao M 2012 Complete genome sequence of a novel porcine epidemic diarrhea virus in south China. J. Virol. 86:10248–10249. 10.1128/JVI.01589-12 22923806 PMC3446595

[18] Kocherhans R Bridgen A Ackermann M Tobler K 2001 Completion of the porcine epidemic diarrhoea coronavirus (PEDV) genome sequence. Virus Genes 23:137–144. 10.1023/A:1011831902219 11724265 PMC7089135

[19] Li B Liu H He K Guo R Ni Y Du L Wen L Zhang X Yu Z Zhou J Mao A Lv L Hu Y Yu Y Zhu H Wang X 2013 Complete genome sequence of a recombinant porcine epidemic diarrhea virus strain from eastern China. Genome Announc. 1(2):e00105-13 e00105-13. 10.1128/genomeA.00105-13 23599287 PMC3630398

[20] Luo Y Zhang J Deng X Ye Y Liao M Fan H 2012 Complete genome sequence of a highly prevalent isolate of porcine epidemic diarrhea virus in south China. J. Virol. 86:9551 9551. 10.1128/JVI.01455-12 22879620 PMC3416124

[21] Wang X-M Niu B-B Yan H Gao D-S Huo J-Y Chen L Chang H-T Wang C-Q Zhao J 2013 Complete genome sequence of a variant porcine epidemic diarrhea virus strain isolated in central China. Genome Announc. 1(1):e00243-12 e00243-12. 10.1128/genomeA.00243-12 23469356 PMC3587950

[22] Wei Z-Y Lu W-H Li Z-L Mo J-Y Zeng X-D Zeng Z-L Sun B-L Chen F Xie Q-M Bee Y-Z Ma J-Y 2012 Complete genome sequence of novel porcine epidemic diarrhea virus strain GD-1 in China. J. Virol. 86:13824–13825. 10.1128/JVI.02615-12 23166239 PMC3503055

[23] Zhao M Sun Z Zhang Y Wang G Wang H Yang F Tian F Jiang S 2012 Complete genome sequence of a Vero cell-adapted isolate of porcine epidemic diarrhea virus in eastern China. J. Virol. 86:13858–13859. 10.1128/JVI.02674-12 23166259 PMC3503114

